# Supplementary material for: Predicting viral sensitivity to antibodies using genetic sequences and antibody similarities
Source: bioRxiv. 2025 Aug 11:2025.08.08.669352. Preprint. [Version 1] doi: 10.1101/2025.08.08.669352 (PMC12363850; doi:10.1101/2025.08.08.669352)
Supplement: Supplement 1 [file NIHPP2025.08.08.669352v1-supplement-1.pdf]

bioRxiv preprint doi: https://doi.org/10.1101/2025.08.08.669352; this version posted August 11, 2025. The copyright holder for this preprint (which was not certified by peer review) is the author/funder, who has granted bioRxiv a license to display the preprint in perpetuity. It is made available under aCC-BY 4.0 International license.

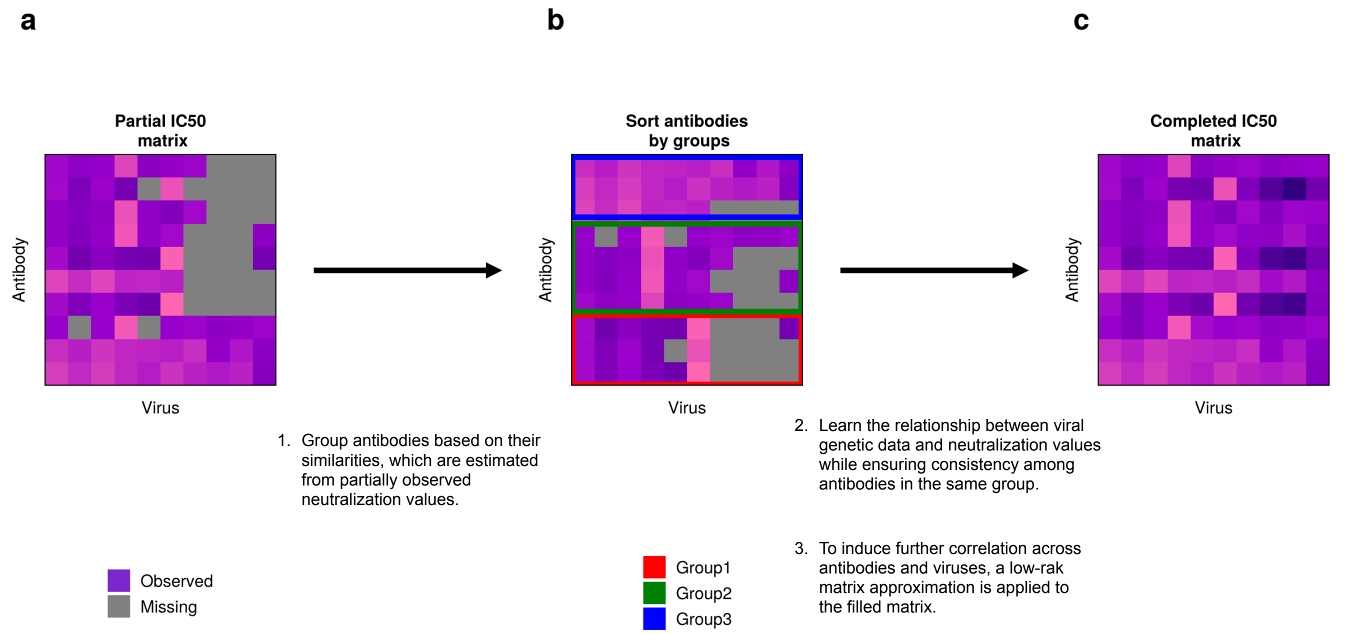

Supplementary Fig. 1. Overview of the neutralization imputation processes a. The partially observed neutralization matrix (columns: viruses, rows: antibodies) is used as input to impute the missing elements (gray scale) of the neutralization matrix. **b**. Schematic representation of grouping antibodies based on the similarity of their neutralization values. Within the same group, neutralization activities are more similar than those between different groups. **c**. Temporarily complete missing values using models that learn the relationship between viral genetic data and neutralization values. We employ the novel grouped learning method, training the model to simultaneously learn the relationships between neutralization values and viral genetic sequences for a group of antibodies that share similar neutralization profiles (Methods). Here, to effectively reduce the number of model parameters and avoid the common over-parametrization issue, we applied a standard dimensional reduction technique to viral sequences and trained the model using the projected sequences. **d**. Obtain the complete neutralization matrix, derived from the low-rank matrix approximation of the temporarily completed matrix from step **c**. The optimal matrix rank was determined based on the distribution of eigenvalues (Methods).

bioRxiv preprint doi: https://doi.org/10.1101/2025.08.08.669352; this version posted August 11, 2025. The copyright holder for this preprint (which was not certified by peer review) is the author/funder, who has granted bioRxiv a license to display the preprint in perpetuity. It is made available under aCC-BY 4.0 International license.

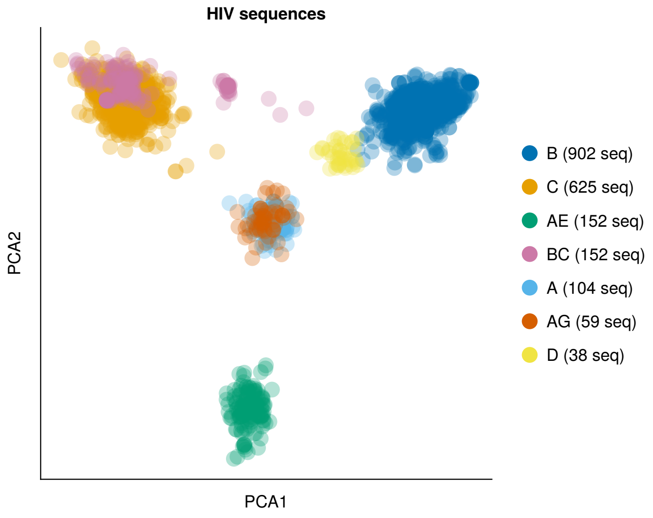

Supplementary Fig. 2. Distribution of HIV sequences across multiple subtypes in the first and second principal modes of the genetic covariance matrix. Each point in the cloud represents a viral sequence projected onto the principal modes of the covariance matrix derived from the one-hot encoded sequences. These principal modes yield projected sequences that are also used to learn the neutralization values. In this visualization, we present only subtypes that include more than 30 viral sequences that are used in our training data. Subtypes such as B, C, A, and AE are mapped to distinct regions in the space.

bioRxiv preprint doi: https://doi.org/10.1101/2025.08.08.669352; this version posted August 11, 2025. The copyright holder for this preprint (which was not certified by peer review) is the author/funder, who has granted bioRxiv a license to display the preprint in perpetuity. It is made available under aCC-BY 4.0 International license.

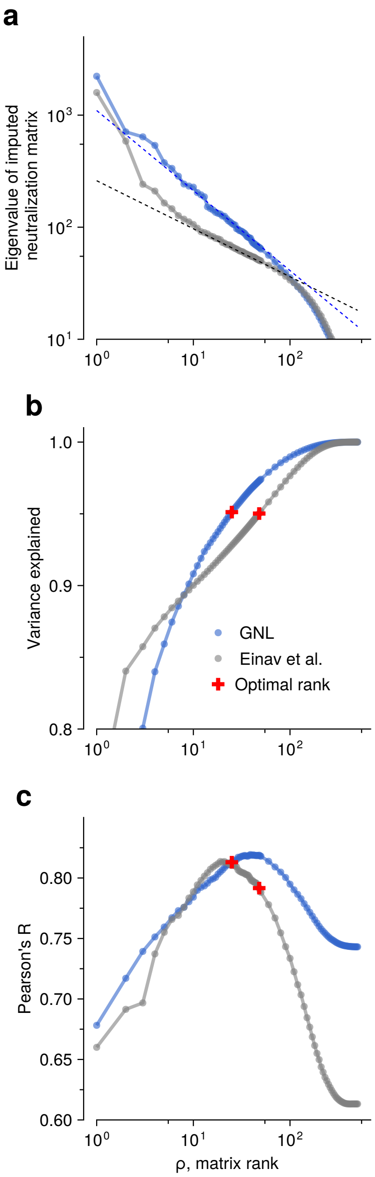

Supplementary Fig. 3. Variance explained and correlation between predicted and true neutralization values as a function of rank. As a typical example, we set the fraction of observed data as 80%, and the validation values were withheld uniformly at random from the CATNAP dataset. **a**, Eigenvalues for the filled neutralization matrices in the GNL and Einav et al. methods. The intermediate ranks of eigenvalues roughly follow a power law. Dashed lines show the power law fit with exponents of $-3/7$ and $-5/7$, respectively. **b**, Profiles of the variance explained for GNL and Einav et al. methods. The "optimal" matrix rank values, defined as the minimum rank at which the explained variance exceeds 95%, are 20 and 45 for the GNL and Einav et al. methods, respectively. **c**, Pearson's R between true and predicted neutralization values shown as a function of rank. Including additional eigenmodes ultimately reduces predictive power. The rate of decrease in Pearson's R using the GNL method is more gradual than that of Einav et al., and it maintains higher values.

bioRxiv preprint doi: https://doi.org/10.1101/2025.08.08.669352; this version posted August 11, 2025. The copyright holder for this preprint (which was not certified by peer review) is the author/funder, who has granted bioRxiv a license to display the preprint in perpetuity. It is made available under aCC-BY 4.0 International license.

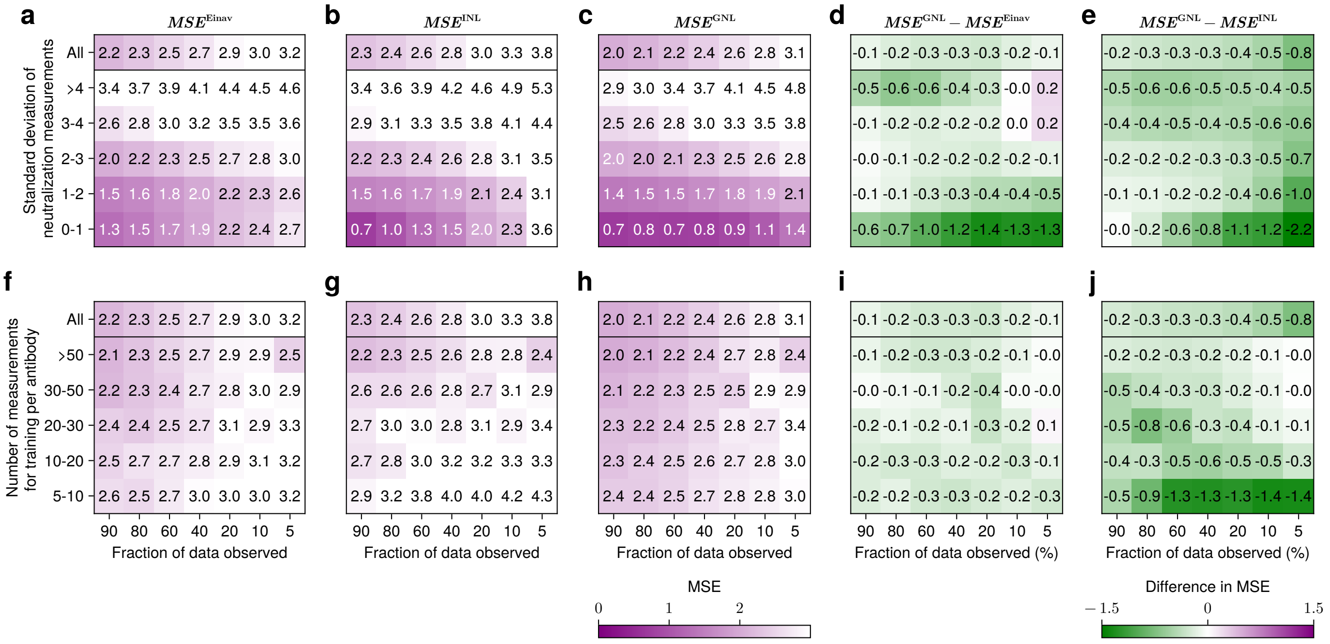

Supplementary Fig. 4. **a–b**, MSE values across seven data observation fractions (5% to 90%) and six classes of the standard deviation of neutralization measures, for the Einav et al. method, Independent Neutralization Learning (INL), and the GNL model. **d–e**, Differences in MSE values between GNL and the other methods. GNL outperforms INL for antibodies with higher variability in neutralization and for lower fractions of observed data (**e**), suggesting that antibody grouping mechanisms aid in learning from variable cases. **f–g**, MSE values across different numbers of training measurements and (**i–j**) their corresponding MSE differences. The grouping method benefits antibodies with fewer observed measurements, suggesting the advantage of grouping for sparsely observed antibody cases (**j**).

bioRxiv preprint doi: https://doi.org/10.1101/2025.08.08.669352; this version posted August 11, 2025. The copyright holder for this preprint (which was not certified by peer review) is the author/funder, who has granted bioRxiv a license to display the preprint in perpetuity. It is made available under aCC-BY 4.0 International license.

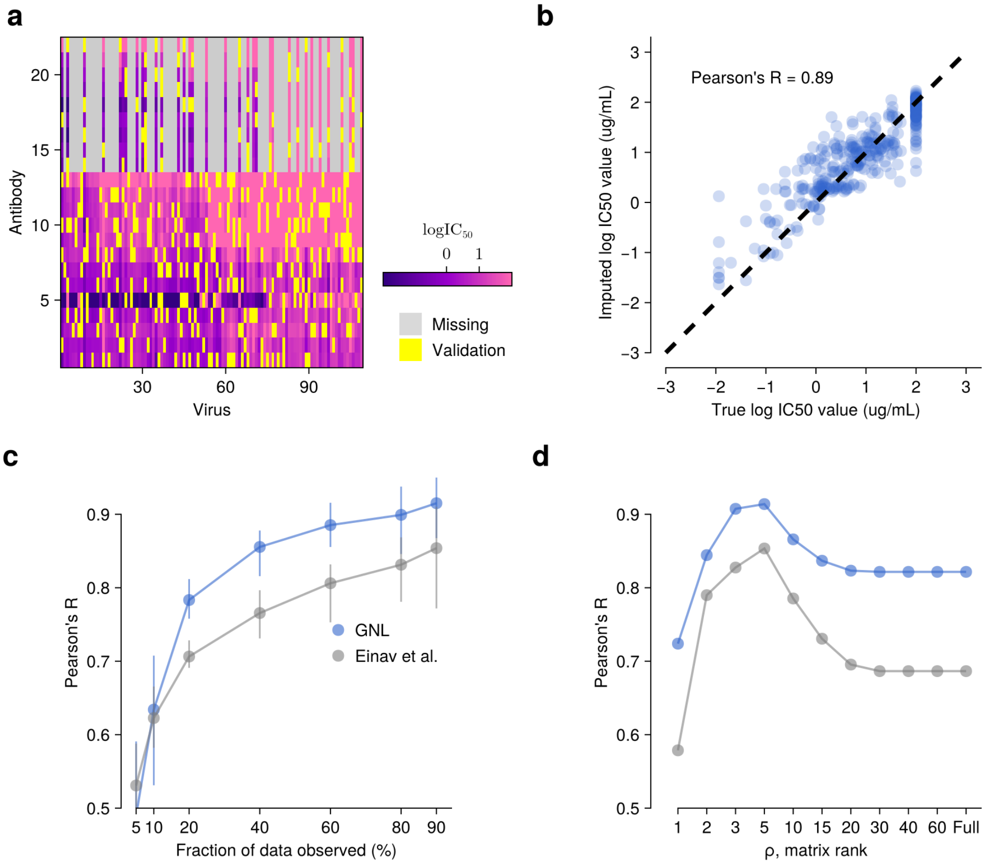

Supplementary Fig. 5. Overall neutralization imputation performance for the individual host. a, Neutralization values are represented with purple, gray, and yellow colors, corresponding to observed, missing, and withheld values for validation, respectively. The withheld elements are chosen uniformly at random across antibodies and viruses. In this analysis, 80% of the total available data is observed. **b**, A comparison of the true withheld neutralization values and the imputed values is shown on the x- and y-axes. Pearson's R value is 0.89. **c**, Accuracy Dependency on the Fraction of Observed Data. The overall accuracy of the GNL method is higher than that of the Einav et al. method (provided by Einav et al.[37]) as the fraction of observed data increases. **d**, Dependency of accuracy on matrix rank ρ. The R values of the GNL method are consistently higher than those of the Einav et al. method across most rank values.
